# Combining Well-Tempered Metadynamics Simulation and SPR Assays to Characterize the Binding Mechanism of the Universal T-Lymphocyte Tetanus Toxin Epitope TT830-843

**DOI:** 10.1155/2021/5568980

**Published:** 2021-07-04

**Authors:** Artur A. M. L. Brandt, Rodrigo N. Rodrigues-da-Silva, Josué C. Lima-Junior, Carlos R. Alves, Franklin de Souza-Silva

**Affiliations:** ^1^Faculdade de Educação Tecnológica do Estado do Rio de Janeiro, Rua Clarimundo de Melo, 847, CEP 21311-281, Rio de Janeiro, RJ, Brazil; ^2^Univeritas-Rio, Rua Marques de Abrantes, 55, CEP 2230-060, Rio de Janeiro, RJ, Brazil; ^3^Fundação Oswaldo Cruz, Instituto de Tecnologia em Imunobiológicos, Laboratório de Tecnologia Diagnóstica, Avenida Brasil, 4365, CEP 21040-900, Rio de Janeiro, RJ, Brazil; ^4^Fundação Oswaldo Cruz, Instituto Oswaldo Cruz, Laboratório de Imunoparasitologia, Avenida Brasil, 4365, CEP 21040-900, Rio de Janeiro, RJ, Brazil; ^5^Fundação Oswaldo Cruz, Instituto Oswaldo Cruz, Laboratório de Biologia Molecular e Doenças Endêmicas, Avenida Brasil, 4365, CEP 21040-900, Rio de Janeiro, RJ, Brazil; ^6^Centro de Desenvolvimento Tecnológico em Saúde, Fundação Oswaldo Cruz, Rio de Janeiro, Brazil; ^7^Universidade Iguaçu, Faculdade de Ciências Biológicas e da Saúde, Rio de Janeiro, RJ, Brazil

## Abstract

Peptide TT830-843 from the tetanus toxin is a universal T-cell epitope. It helps in vaccination and induces T-cell activation. However, the fine molecular interaction between this antigen and the major histocompatibility complex (MHC) remains unknown. Molecular analysis of its interaction with murine MHC (H-2) was proposed to explore its immune response efficiency. Molecular dynamics simulations are important mechanisms for understanding the basis of protein-ligand interactions, and metadynamics is a useful technique for enhancing sampling in molecular dynamics. SPR (surface plasmon resonance) assays were used to validate whether the metadynamics results are in accordance with the experimental results. The peptide TT830-843 unbinding process was simulated, and the free energy surface reconstruction revealed a detailed conformational landscape. The simulation described the exiting path as a stepwise mechanism between progressive detachment states. We pointed out how the terminus regions act as anchors for binding and how the detachment mechanism includes the opening of *α*-helices to permit the peptide's central region dissociation. The results indicated the peptide/H-2 receptor encounter occurs within a distance lesser than 27.5 Å, and the encounter can evolve to form a stable complex. SPR assays confirmed the complex peptide/H-2 as a thermodynamically stable system, exhibiting enough free energy to interact with TCR on the antigen-presenting cell surface. Therefore, combining in silico and in vitro assays provided significant evidence to support the peptide/H-2 complex formation.

## 1. Introduction

*Clostridium tetani* is a pathogenic gram-positive bacterium causing tetanus. This bacterium can be isolated from soil and animal intestinal tracts and, as such, can contaminate many surfaces and substances [1]. *Clostridium tetani* is a restricted anaerobic bacillus, a spore producer that allows it to survive under aerobic conditions and produces an exotoxin, a tetanus toxin, that attaches itself to the system nervousness, provoking the symptomatology of the disease [2].

The tetanus toxin is a peptidase tentoxilysin (Clan MA, M27 family) [3], which is produced by the anaerobic spore-forming bacteria *C. tetani* as a single-chain polypeptide that is 1315 amino acids (aa) in length [4]. The protein is endogenously processed to yield the 52 kDa light chain (457 aa) and the 98 kDa heavy chain (858 aa). A disulfide bridge forms a dimeric protein of 150 kDa, showing a toxic property due to its ability to bind to specific membrane receptors on presynaptic motor nerve cells. Therefore, this interaction causes interneuron discharge inhibition affecting the motor and autonomic nervous system [5–8].

The heavy chain fragment of the tetanus toxin is marked by its antigenic and immunogenic properties [9], which is related to effective, specific, and safe antitoxin vaccines [10]. This fragment stands out by its ability to induce the production of antibodies when used as a fusion partner for foreign antigens [11–13]. Tetanus toxin fragment adjuvant potency is related to the presence of promiscuous T-lymphocyte epitopes in the protein [14]. The tetanus toxoid protein contains several T-lymphocyte epitopes (p2, p21, p23, p30, and p32), which have been extensively studied. They were demonstrated to be universally immunogenic T-cell epitopes in both mice and humans. Among these peptides stands the p2 epitope TT830-843 (QYIKANSKFIGITE), located at the protein C-terminus region, which can bind major histocompatibility complex (MHC) proteins [14–16].

As a linear T-lymphocyte epitope, the TT830-843 sequence is related to T-lymphocyte activation and is used to improve vaccine potential. For example, it has been reported that a TCR (T-cell receptor) set can recognize the TT830-843 epitope presented by DRB1∗1302 (human MHC class II DRB1∗1302 allele). It indicates that TCRs with distinct CDR3s (complementary-determining region 3) in the conserved V beta-chain may not differ in how they recognize the ligand and may provide new insights into understanding the TCR/peptide/MHC supramolecular complex formation [17].

The TT830-843 sequence has been used as an adjuvant universal epitope of the immune system in different vaccine platforms due to its significant properties as a T-lymphocyte helper epitope. This peptide fragment enhances antigenicity and the overall immune response. In this context, the use of the TT830-843 epitope as a peptide conjugated to antigenic epitopes of interleukin-13 has been proposed to increase the efficacy of vaccine protective humoral immunity in murine asthma models [18]. Another example is type I diabetes in mice whose expression of the insulin B:9–23 sequence is required to develop anti-insulin autoimmunity [19, 20]. The sensibilization approach using the TT830-843 epitope as an adjuvant to modify insulin B:9–23, intranasally induced, provided significant suppression of diabetes [21]. Rodrigues-da-Silva et al. [22] demonstrated the fusion of the TT830-843 sequence in a synthetic construction against *Plasmodium vivax*-enhanced specific T- and B-cell responses to a vaccine candidate.

**Figure 1:**
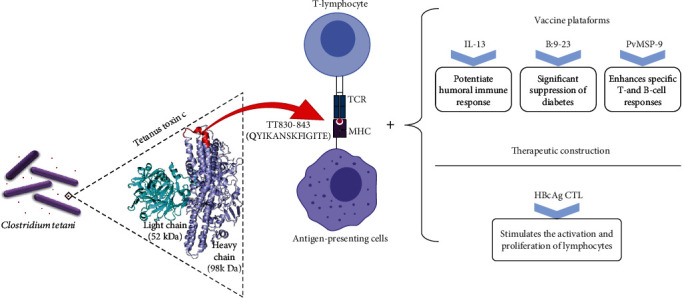
Schematic model of the action of the tetanus toxin-derived peptide TT830-843. Tetanus toxin is produced by the bacteria *C. tetani*. The heavy chain fragment of tetanus toxin is marked by its antigenic and immunogenic properties. The protein contains several T-lymphocyte epitopes, in particular the peptide TT830-843. The epitope binds to MHC proteins and is exposed on the surface of antigen-presenting cells. The TT830-843/MHC interacts with the T-cell receptor and activates a T-lymphocyte response. Used as an adjuvant universal epitope, TT830-843 can enhance the immune response in vaccine platforms and therapeutic construction.

In addition, a study of the immunogenicity of hepatitis B virus epitope-based polypeptides to trigger a specific HLA I-restricted (human leukocyte antigen class I) CD8^+^ T-lymphocyte response was proposed by constructing mimetic peptides based on the introduction of the TT830-843 epitope to strengthen the T-lymphocyte response. This therapeutic construction improved the induction of CD8^+^ CTL-mediated (cytotoxic T-lymphocyte) cytotoxicity in HLA-A2^+^ human peripheral blood lymphocytes [23]. In Figure 1, we present a schematic model of the action of the tetanus toxin-derived peptide TT830-843.

Even with all these data showing the enhanced immune response provided by the TT830-843 fragment, little is known regarding to the molecular mechanisms underlying this phenomenon. Bioinformatics tools can explain the molecular foundations of immunity and validate potential epitopes for vaccine candidates [24]. However, current *in silico* computational methods for the validation and investigation of T-cell epitope interactions are far from satisfactory and vary in degree of accuracy. For example, most of these current methods confirm experimentally predicted binding to MHC molecules of most peptides predicted, but with only ~10% of those shown to be immunogenic [25]. Therefore, we explore the feasibility of combining an *in silico* computational approach and an *in vitro* physicochemical assay.

In this work, the association of an *in silico* and a physicochemical approach was proposed to access the details of the molecular interaction between the TT830-843 peptide and the murine MHC (H-2) receptor. An *in silico* computational approach, well-tempered metadynamics (WTMetaD), was applied to enhance the sampling of the free energy configurational space. WTMetaD introduces a bias potential that acts on a select number of degrees of freedom, called collective variables (CVs) [26]. Additionally, the peptide binding affinity to the H-2 protein and the kinetic parameters of this association were accessed by physiochemical assays. Surface plasmon resonance (SPR) was used for this purpose, monitoring the changes in the refractive index at the surface of a carboxyl sensor chip (COOH5) [27].

## 2. Materials and Methods

### 2.1. Molecular Dynamics (MD) and Well-Tempered Metadynamics (WTMetaD)

MD simulation is an important method for understanding the physical basis of biological macromolecule structure and function. MD became an especially useful computational technique to simulate biological processes inside the cells [28]. With the understanding of the system's internal motions and their implications, questions concerning particles' conformational changes as a function of simulation time can be explored. Therefore, MD simulation results can be used to address questions about specific properties of biological entities that would be more difficult to address in real systems [28].

However, with ongoing advances in computational simulations, standard MD methods often fail to adequately explore the configurational space to accurately evaluate proteins' thermodynamics and kinetic properties [29]. Standard MD simulation requires a large amount of computational time to run and provide meaningful data for analysis. This situation occurs since high free energy barriers separate the relevant equilibrium configuration states, and the simulation tends to revisit the same energy minimum. For example, in rare events, like peptide ligands unbinding from MHC proteins, the system is trapped in configurational space local regions over the simulation time scale. This behavior is because there are significant high free energy barriers to be overcome, and the simulation cannot move from one stability state to another, i.e., from the binding state to the unbinding state. Therefore, in this case, standard MD simulations cannot reproduce biological processes in a feasible computational time [29]. Enhanced sampling techniques can be applied to address this issue and successfully perform biological rare phenomenon simulations.

WTMetaD belongs to a class of techniques that enhance the sampling of certain degrees of freedom, known as collective variables (CVs). WTMetaD facilitates the sampling of the configurational space by introducing a bias potential that acts on this selected number of degrees of freedom [26]. This method helps to accelerate computer simulations by adding a history-dependent potential to the system. One can say WTMetaD is a standard dynamic simulation in which it is imposed a harmonic restraint on a set of CVs. Therefore, WTMetaD can obtain accurate results in a relatively short simulation time [30].

WTMetaD is successfully applied to simulate rare events, predict binding affinity, and investigate intermolecular interactions occurred during the simulations. Additionally, enhanced sampling approaches permit to reconstruct the free energy surface (FES) associated with the protein interaction dynamics as a function of CVs [26, 30, 31]. WTMetaD operates a dimensional reduction of the degrees of freedom of a system. Energetic landscapes obtained from WTMetaD can be used to understand the intermolecular interactions occurring in the metastable states visited by the system. It also helps to explore many possible transition pathways between different free energy minima. Enhanced sampling technics have been applied to obtain meaningful data to study the dissociation mechanisms in protein complexes [31], becoming an important *in silico* method for biological research.

### 2.2. Initial Molecular Structure Preparation for Peptide/H-2D^b^ Complex

MHC class I starting structure was obtained from the Protein Data Bank (PDB) [32]. PDB entry 1jpf was used as a template to build the peptide/H-2D^b^ complex structure. PDB entry 1jpf has a resolution of 2.18 Å and represents the most extended peptide sequence (with 11 amino acids) whose structure has been determined for the H-2D^b^ haplotype [33]. Peptide TT830-843 was modeled into the H-2D^b^ receptor binding groove by using the psfgen package in VMD [34] and submitted the resulting coarse starting model to a proper refinement protocol.

The H-2D^b^ haplotype (receptor) structure in complex with the peptide TT830-843 (ligand) was submitted to the Rosetta FlexPepDock web server [35] for the refinement of the coarse starting model. Rosetta FlexPepDock is implemented in the Rosetta modeling suite framework [36] and performs a flexible peptide docking refinement protocol allowing full flexibility for the peptide and receptor sidechains. A standard refinement FlexPepDock protocol was applied (with default options selected) to model the peptide/H-2D^b^ complex. The FlexPepDock web server optimized the peptide conformation within the H-2D^b^ protein cleft, carrying out two hundred independent simulations: the first hundred in high-resolution mode and the remainder by applying the low-resolution preoptimization step, with a high-resolution posterior refinement. Resulting models were ranked based on Rosetta's generic full-atom energy score [36], measuring the peptide/H-2D^b^ complex's binding affinity. The best-ranked atomic coordinate pose was used (the lowest energy structure) (see Table S2 in Supplementary file 1) as the initial structure for WTMetaD simulation. The Rosetta FlexPepDock web server also calculates bb-RMSD (RMSD calculated for all peptide backbone atoms) from the starting conformation. Complex submitted to Rosetta FlexPepDock was obtained from a mutation in an existing PDB structure; consequently, the bb-RMSD calculated during the refinement does not correspond to a deviation from a crystallographic structure. Therefore, these conditions explain why we considered the Rosetta energy score to choose the initial structure for WTMetaD simulation. Top 10 resulting models obtained from the Rosetta FlexPepDock webserver are shown in Figure S2 of Supplementary file 1. The stability of the peptide initial structure obtained from the Rosetta FlexPepDock webserver is demonstrated in Figure S3 of Supplementary file 1.

### 2.3. Simulation Setup

All simulations were performed using NAMD 2.13 [37] with the CHARMMM27 force field [38]. Electrostatic interactions were evaluated using the Particle Mesh Ewald (PME) algorithm with a grid spacing of 1 Å. Nonbonded interactions were truncated using a cutoff of 12 Å and a switching function starting at a radius of 10 Å. Protein-peptide complex was immersed in an orthorhombic box using periodic boundary conditions. System was inserted within a 10 Å (for *x*- and *y*-axes) and 40 Å (along the *z*-axis) layer of water molecules, containing around 13,200 TIP3P water particles [39]. The complex structure was oriented to keep the peptide's exiting direction aligned with the *z*-axis. To neutralize the system, Na^+^ counterions were added. The simulations ran in physiological pH, and the protonation states of the protein and the peptide residues were selected to conform to this physiological pH range. Therefore, the protonation states of all residues were assigned according to the pKa values of their side chains using the psf builder [34]. In particular, the protonation states for histidine residues were set as HSE (with their *ϵ* nitrogen protonated). Equations of motion were integrated using a velocity Verlet integration algorithm with a timestep of 2 fs in an NPT ensemble. The SHAKE algorithm was used to constrain covalent bonds. Energy minimization of the starting structure involved 15000 steps of the steepest descent method. Next, the system was equilibrated in a three-stage protocol: (i) heating up from 10 K to 310 K by increasing the temperature by 10 K for every 100 steps, and with the CA atom positions restrained using a harmonic potential with a force constant of 0.25 kcal/mol/Å^2^; (ii) 1 ns of water equilibration at 310 K, applying harmonic restraints to the CA atom positions (0.25 kcal/mol/Å^2^ force constant) to allow water molecules to fully envelope the complexes; and (iii) peptide equilibration (1 ns of MD simulation) at 310 K, in which the peptide was free, keeping harmonic restraints only for CA atoms of the H-2D^b^ protein (0.25 kcal/mol/Å^2^ force constant).

**Figure 2:**
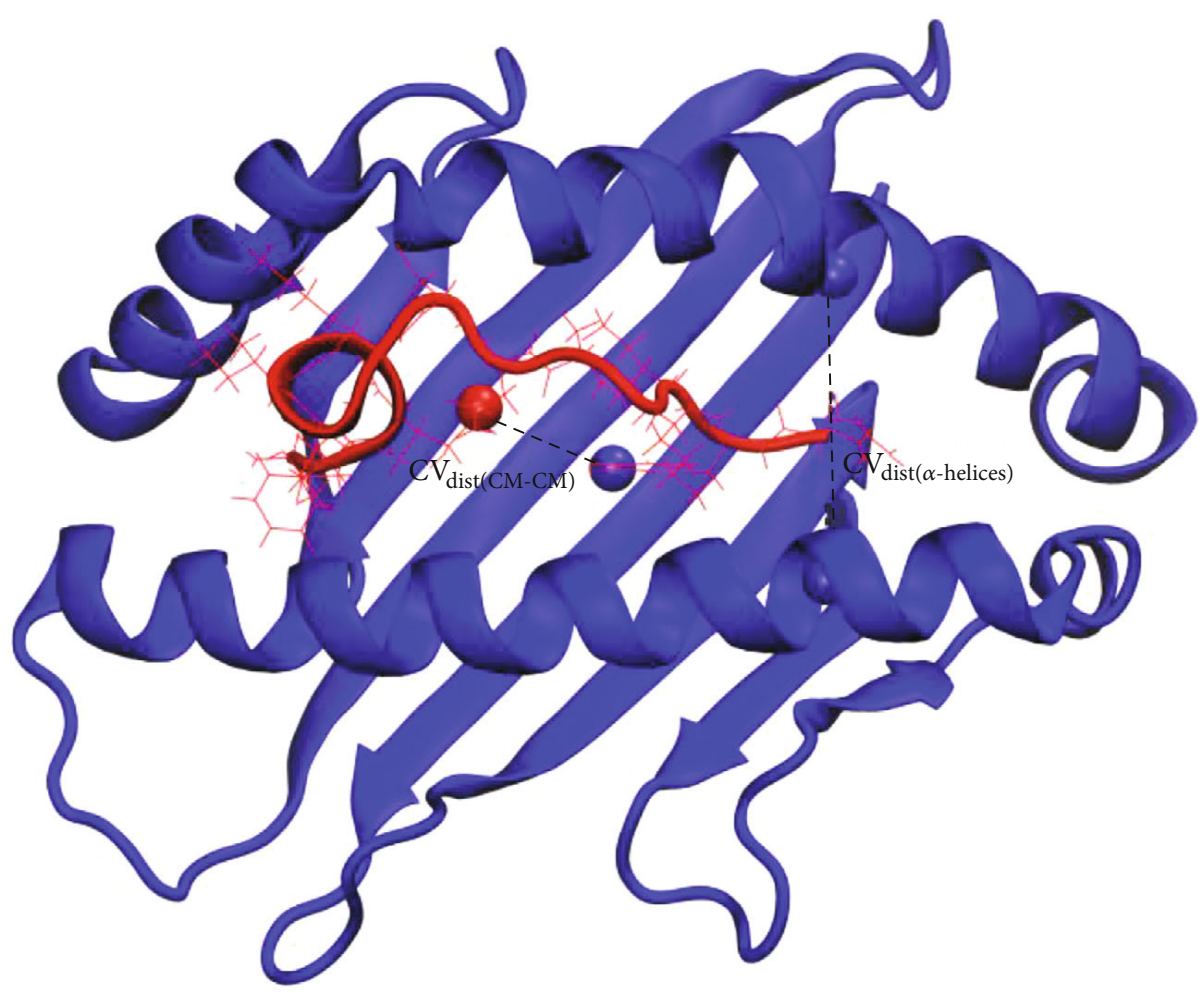
Collective variables chosen for WTMetaD simulation. Black dotted lines represent both CVs. CV_dist(CM-CM)_ was defined as the distance between the centers of mass of the peptide TT830-843 and the H-2D^b^ protein. CV_dist(*α*-helices)_ was defined as the distance between the centers of mass of the two *α*-helical segments in the N-terminus binding cleft (group of residues 56-70 and residues 155-175). CV_dist(CM-CM)_ varied from 8 Å to 38 Å, and CV_dist(*α*-helices)_ varied from 10 Å to 28 Å.

For the simulations in the NPT ensemble, the temperature was maintained at 310 K by Langevin dynamics, and the pressure was kept constant (1 atm) by the Langevin piston method. During WTMetaD simulation, the peptide and the two *α*-helical segments that flank the N-terminus peptide binding region (*α*1-helix residues 56-70 and *α*2-helix residues 155-175) were free to move, while other CA atom positions were restrained using a harmonic potential with the same force constant of 0.25 kcal/mol/Å^2^. WTMetaD simulations were performed for 16 ns, with Gaussians height set at 0.05 kcal/mol, a new hill added to the WTMetaD potential every 0.2 ps, and a biasing potential set at 3000 K. Collective variables (CVs) were defined as the distance between the centers of mass of the peptide and the H-2D^b^ protein, named CV_dist(CM-CM)_, and the distance between the centers of mass of the two N-terminus binding cleft *α*-helical segments (group of residues 56-70 and residues 155-175), named CV_dist(*α*-helices)_ (see Figure 2 for the visualization of these CVs). CV_dist(CM-CM)_ varied from 8 Å to 38 Å with a Gaussian width of 1.25331 Å, and CV_dist(*α*-helices)_ varied from 10 Å to 28 Å with a Gaussian width of 0.626657 Å. Simulation-saved potential mean force (PMF) maps every 1 ps.

### 2.4. Surface Plasmon Resonance (SPR)

SPR is an optical biosensing method that can directly determine the biomolecular interaction kinetic parameters [27]. When polarized light impinges through a prism upon a sensor chip with a thin metal film on top, it is reflected by the metal film acting as a mirror. Depending on the angle of incidence, the light's energy is enough to create electron waves (or plasmons) on the metal surface (usually gold) film. On changing this angle, a decrease in the reflected light intensity detects the surface plasmon. The angle correspondent to the minimum intensity of the light reflected is called the resonance angle. Refractive index near the metal surface changes when it accumulates mass adsorbed on the thin gold layer. Changes in the refractive index imply a shift in the angle at which the minimum intensity of the light reflected is observed. Therefore, SPR biosensors monitor the interaction between a mobile molecule in the solution (analyte) and a biospecific partner immobilized on the metal surface by evaluating the changes in the resonance angle shift.

SPR assays were performed on carboxyl sensor chips (COOH5; Pall Fortebio LLC, Fremont, CA, USA) and performed on a Pioneer Sensing Optical Transduction Biosensor (ICx Nomadics Inc., Oklahoma City, OK, USA). The recombinant soluble dimeric mouse H-2D^b^:Ig (DimerX) protein was purchased from BD Biosciences (San Jose, CA, USA). Briefly, upon immobilization, the biosensor surface was activated using a 1 : 1 solution of 1-ethyl-3-(3-dimethylaminopropyl) carbodiimide (EDC)/N-hydroxysuccinimide (NHS) for 5 minutes. The DimerX protein (0.1 *μ*g/*μ*L) was immobilized at a constant flow rate (50 *μ*g/*μ*L for 4 min) and injection of 1 M ethanolamine HCL pH 8.5 to block the remaining carboxyl groups before the binding assays. Ligand binding (synthetic epitope) to the immobilized receptor (DimerX) was evaluated using the peptide (31 *μ*M), at a constant flow rate (10 *μ*L/min for 4 min) in PBS with 0.1% dimethylsulfoxide (DMSO; Sigma-Aldrich Chemical Co., St Louis, MO, USA). Binding assays were recorded in real time using a sensorgram, in which the changes in SPR angle (*θ* spr) were measured as arbitrary units of resonance (RU). UR data were analyzed after subtraction of reference channel UR values using QDAT software (ICx Nomadics Inc., Oklahoma City, OK, USA).

### 2.5. Tetanus Toxin Epitope Sequence

Tetanus toxin epitope TT830-843 was synthesized using fluorenylmethoxycarbonyl-protected amino acids at a 95% purity rate by WatsonBio Sciences (WatsonBio Sciences, Houston, TX, USA). The peptide was dissolved in 10% of DMSO solution prepared with ultrapure water (*v*/*v*) and stored at -20°C before using in surface plasmon resonance (SPR) assays.

## 3. Results

H-2D^b^ protein is an MHC class I haplotype protein of C57BL/6 mice. H-2D^b^ protein is related to antigen presentation to T-lymphocytes expressing CD3/TCR and CD8 proteins [40]. This genetically modified mouse strain is of the most widely used models for human diseases and one of the most used for preclinical vaccine assays [41]. Therefore, this work studied the interatomic interactions within the peptide/H-2D^b^ complex to reveal relevant characteristics that promote complex stability in antigen-presenting cells.

Biological processes, like peptide interaction with H-2D^b^ receptors, rely on molecular organization and recognition phenomena. Peptide-binding process simulations require the entry and accommodation of the ligand within its binding site. This process has both enthalpic and entropic components and implies changes in each counterpart structure and dynamics [42]. However, when the ligand is outside the binding site surrounded by the solvent, it can adopt many different states. Therefore, we opted to perform WTMetaD simulation with the peptide starting from their bound state, thus simulating the unbinding process, i.e., the peptide exiting from the H-2D^b^ protein (see Supplementary files 2 and 3 for movies presenting the simulation evolution).

Initially, different CVs were tested to examine the behavior of the peptide unbinding process. Tests were performed to define which set of CVs would best simulate the peptide detachment. After the CV test stage, the most appropriate CVs were defined as the distance between the centers of mass of the peptide and H-2D^b^ protein, named CV_dist(CM-CM)_, and the distance between the centers of mass of the two *α*-helical segments in the N-terminus binding cleft (the group of residues 56-70 and residues 155-175), named CV_dist(*α*-helices)_ (see Figure 2 for visualization of the CVs chosen). The CVs chosen allowed WTMetaD simulation to overcome the energy barriers between bound and unbound states. Other tested CVs were not able to properly simulate the peptide exiting. These CVs could distinguish between bound and unbound states and describe the relevant intermediate states along the peptide exiting from the cleft. CV_dist(*α*-helices)_ describes the motion of the N-terminus peptide binding region, opening to permit the movement of the peptide from binding to the unbinding state. Two *α*-helical segments that flank the peptide N-terminus binding region (residues 56 to 70 of the *α*1-helix and residues 155 to 175 of the *α*2-helix) reveal significant differences in conformational flexibility compared to the C-terminus peptide binding region (which is more flexible) [43]. Therefore, the CV_dist(*α*-helices)_ choice considered that the N-terminus binding region adopts a more conserved and more stable structure.

In WTMetaD, the bias potential added to the system decreases during the simulation. Therefore, WTMetaD offers an estimator of the FES that converges to the exact result in the long time limit [26, 30].

CV trajectories display the peptide transition from the bound to unbound states. Trajectories were inspected to check whether bound and unbound states were sampled. During the simulation, WTMetaD filled the first main minimum (bound state) before filling the second one (free unbound state). The free energy of dissociation (Δ*G*_diss_), computed as function of the simulation time, was evaluated to validate the convergence. Δ*G*_diss_ is the free energy difference between bound and unbound states.

**Figure 3:**
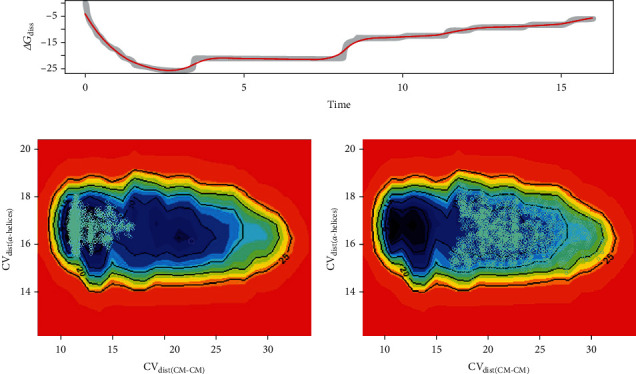
Simulation convergence. (a) Δ*G*_diss_ values (free energy difference between bound and unbound states) plotted as a function of WTMetaD simulation time in nanoseconds (ns). The simulation converged at 16 ns. In this period, the binding and unbinding states were completely sampled. *ΔG*_diss_ was measured in kcal/mol. (b) In the background, the 2D isosurface represents the FES, as described in Figure 4. Blue points indicate the sampling in CV space for the initial 6 ns, showing that the simulation initially explored the peptide binding state. (c) Blue points indicate the sampling in CV space between 6 ns and 16 ns, indicating the simulation completely explored the peptide unbound state.

Figure 3(a) shows the Δ*G*_diss_ decreasing due to the transfer of the peptide to the unbound *z* state, forming stable PMFs after 12.5 ns. Considering the PMFs obtained between 12.5 ns and 16 ns, the distribution of the calculated Δ*G*_diss_ indicated its convergence, having a mean value of 8.23 kcal/mol. Therefore, convergence was successfully reached at 16 ns, with the peptide in the unbound state. The FES was reconstructed from this point.

Additionally, Figures 3(b) and 3(c) show how the simulation sampled the FES during the simulation. In Figure 3(b), the figure shows the FES exploration during the first 6 ns, in Figure 3(c), the exploration after 6 ns. In the first 6 ns, the blue points indicate how the system explored the FES in CV space. Initially, the simulation explored the peptide binding state. After 6 ns, the simulation initiated the peptide unbound state exploration, completing this process in 16 ns. Therefore, the simulation explored the FES binding and unbinding states, and the PMF obtained at the end of this period is suitable for reconstructing the system's free energy landscape.

Some structural properties were used to determine whether the peptide unbound state was achieved. Along with the WTMetaD evolution, properties like hydrogen bonds (H-bond), intermolecular interaction energy (electrostatic and Lennard-Jones terms), and SASA (surface accessible solvent area) were used to evaluate the disruption of the peptide-receptor interaction. SASA quantified the amount of exposure of the peptide to the solvent. After the peptide exit, SASA values stabilized at a rate corresponding to the peptide fully exposed to solvent. On the other hand, H-bond and intermolecular interaction energy decreased with the simulation progress, approaching zero. The evolution of these properties indicates the peptide moved away from the H-2D^b^ receptor, achieving a fully hydrated state (see Figure S1 in Supplementary file 1). Therefore, when FES was reconstructed, the peptide was in a completely unbound state.

Persistence of H-bond interactions formed between the peptide and the H-2D^b^ receptor was evaluated.  Table 1 indicates the persistence of H-bond interactions formed along the peptide exiting. WTMetaD trajectory across the simulation frames was analyzed to identify all H-bond interactions formed during this process. In Table 1, the third column's value computes the percentage of the total number of H-bond interactions formed during the simulation. First and second columns represent residues from the peptide and the H-2D^b^ receptor, respectively, that participate in a selected H-bond interaction. Such an analysis provides a measurement of specific H-bond interactions and persistence and can give a sense of their relevance to stabilize the peptide/H-2D^b^ complex formation.

**Figure 4:**
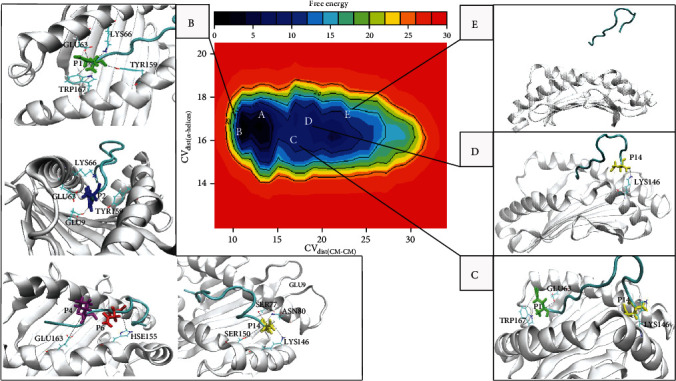
Bidimensional representation of the dissociation free energy surface (FES). This landscape was reconstructed using WTMetaD. Isosurfaces were obtained as a function of CVs, displaying the free energy of dissociation for each pair of CV_dist(CM-CM)_ and CV_dist(*α*-helices)_. Both CVs were measured in angstrom and free energy in kcal/mol. Legends A, B, C, D, and E represent the five FES stable basins. In boxes B, C, and D, the most relevant H-bond interactions formed during WTMetaD are described. Label B indicates the peptide docked position and label E the unbound state.

Peptide unbinding pathway from the H-2D^b^ receptor was investigated to understand the atomistic and molecular details of the interaction. WTMetaD simulation reproduced the unbinding event and showed that the peptide progressively moved away from the cleft to a fully hydrated state. FES exhibits five stable basins, separated by high energy barriers (transition states). Stable basins were marked as A, B, C, D, and E in Figure 4.

Initially, the peptide was exposed to continuous rearrangements seeking favorable interactions, implying a transition from basins A to B. Consequently, in basin B, the complex adopts a new energy conformation that is ~1.15 kcal/mol lower in energy than that in basin A.

The deepest minimum, basin B, corresponds to the peptide in its binding site, in which some persistent H-bond interactions with H-2D^b^ occurred (see Table 1). In this basin, N-terminus peptide residue P1 formed an H-bond network with residues GLU63, LYS66, TYR159, and TRP167 in the cleft. Additionally, the peptide formed relevant H-bond interactions between residue P2 and residues GLU9, GLU63, and LYS66. On the other hand, the C-terminus peptide residue P14 interacted with the *α*2-helix through a significant H-bond interaction network, mainly with residue LYS146. Some other relevant interactions between the peptide's central part and H-2D^b^ receptor were observed, namely, interactions with residues P4 and P6 (see Figure 4, box B).

The third energy basin (basin C) is ~7.31 kcal/mol higher in energy than basin B. B-to-C transition occurred after opening of the *α*-helices, and it might account for the dissociation of the peptide's central part from H-2D^b^. While the simulation was filling basin C, the interactions established between the N-terminus peptide residue P1 and residues GLU63 and TRP167 were conserved. Moreover, the C-terminus peptide residue P14 still maintained interaction with residue LYS146 of the *α*2-helix. Therefore, at this stage of the undocking pathway, the peptide was moved through the cleft of H-2D^b^. The peptide stabilized at a position in which P1 maintained the H-bond with residue GLU63 of the *α*1-helix and residue TRP167 of the *α*2-helix and in which P14 maintained H-bond interaction with residue LYS146 of the *α*2-helix (see Figure 4, box C). WTMetaD overcame this energy barrier and pushed out the system from basin B to basin C. During the B-to-C transition, distance between *α*-helices reached values ~18 Å (CV_dist(*α*-helices)_) to facilitate the dissociation of residues P2, P4, and P6 from the cleft.

Basin D was reached just after the peptide N-terminus region dissociation. In this metastable state, only C-terminus peptide residue P14 interacted with the receptor, maintaining H-bond interactions with the *α*2-helix of the H-2D^b^ protein, mainly with residue LYS146. After this stage of the undocking pathway, the ending of these H-bond interactions permitted the completed disruption of the peptide/H-2D^b^ complex (see Figure 4, box D).

At the last minimum (basin E), the distance between *α*-helices decreased to values close to 16 Å (CV_dist(*α*-helices)_), and the distance between centers of mass of H-2D^b^ and the peptide achieved ~22.5 Å in CV_dist(CM-CM)_ (see Figure 4, box E). It indicated that the peptide reached the fully hydrated state. This state corresponds to the complete disruption of all interactions between the peptide and the H-2D^b^ receptor. At the end of the undocking pathway, the free energy of dissociation calculated as the difference between the energies in basins B (docking position) and E (undocking position) had a mean value of 8.23 kcal/mol. The gif animated image in Supplementary file 4 shows the projection of the FES plot of the configurations in CV space sampled during the WTMetaD simulation.

**Figure 5:**
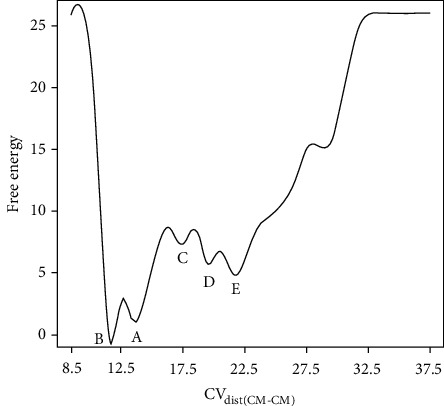
Free energy of dissociation projected onto the CV_dist(CM-CM)_. The figure projects the FES onto the CV_dist(CM-CM)_ by integrating the Boltzmann factor over the CV_dist(*α*-helices)_. Legends A, B, C, D, and E represent the basins described in Figure 4. CV_dist(CM-CM)_ was measured in angstrom and the free energy in kcal/mol.

Time evolution of CV_dist(CM-CM)_ mostly describes the peptide exiting from the H-2D^b^ receptor. Therefore, the FES was projected onto the CV_dist(CM-CM)_ coordinate to measure each basin's free energy value on this surface. In Figure 5, the projection was plotted by integrating the Boltzmann factor over the CV_dist(*α*-helices)_ (Equation (1)). This projection considered the energy values for each pair of CVs [44]. Free energy values for basins A, B, C, D, and E are 1.15, 0, 7.31, 5.72, and 5.10 kcal/mol, respectively.

(1) $$\begin{array}{c} -\beta \omega_{\gamma }\left(Ƶ\right)=\ln\frac{\int e^{-\beta \omega \left(Ƶ,Ƴ\right)}dƳ}{\int e^{-\beta \omega \left(Ƶ,Ƴ\right)}dƵdƳ}. \end{array}$$

In Equation (1), *Ƴ* = CV_dist(*α* − helices)_, *Ƶ* = CV_dist(CM − CM)_, and *β* = 1/*k*_*b*_*T*, where *k*_*b*_ is the Boltzmann constant and *T* = 310 K, *ω* is the function that gives the free energy for each pair of (*Ƶ*, * Ƴ*), and *ω*_*Ƴ*_ is the same function but fixing the value of * Ƴ*.

Binding contacts were monitored by analyzing the intermolecular interactions between the peptide and the cleft of H-2D^b^. The “half-lives” of the interaction energy between residues from the peptide and the H-2D^b^ receptor were monitored. The first half-life was defined as a factor of the simulation time required for the interaction energy to reduce 50% of its initial value. In the same way, the second half-life is the factor required to reduce 75% of the initial value. Initial interaction energy contributions per residue are different. Therefore, half-life factors were weighted and rescaled according to Equation (2) to have values between 0 and 1.

(2) $$\begin{array}{c} x_{\text{new}}=\frac{x-x_{\min}}{x_{\max}-x_{\min}}. \end{array}$$

**Figure 6:**
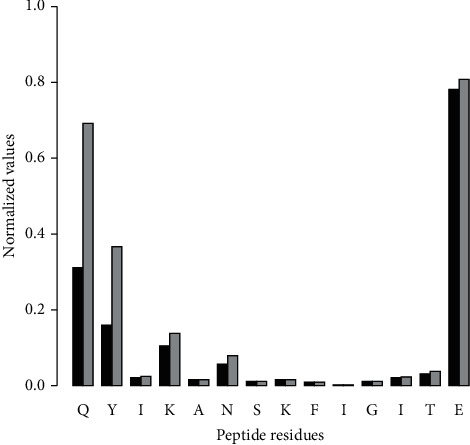
First and second half-lives of the nonbonded interaction energy. Nonbonded interaction energy, computed as electrostatic and Lennard-Jones terms, was evaluated to obtain the first and second half-life factors. First half-life (black bar) is a factor of the simulation time percentage required for the peptide to lose half of its initial intermolecular interaction energy. Second half-life (gray bar) is a factor of the simulation time percentage necessary for the peptide to lose 75% of its initial interaction energy. Factors were rescaled to have values between 0 and 1, according to their initial interaction energy. Values close to 1 indicate a long-lasting interaction between residues and the receptor; values close to 0 indicate a short-lived interaction with the receptor. The figure displays the peptide residues in the one-letter code.

In Equation (2), *x*_new_ is the new weighted initial energy calculated for each peptide residue, *x* is the original value of each residue's initial energy, and *x*_min_ and *x*_max_, respectively, are the minimum and maximum values of the set of initial energies. In Figure 6, the half-life factors indicate the residues of the peptide that play an important role in the unbinding trajectory.

**Figure 7:**
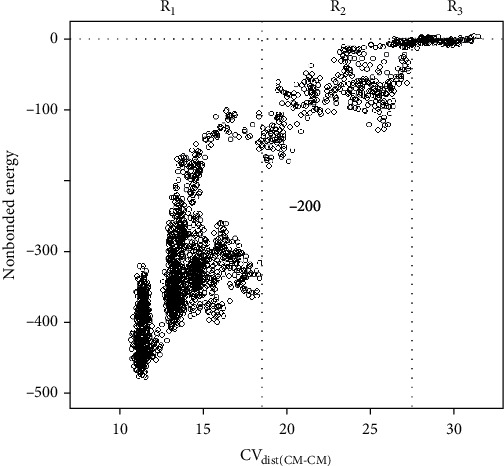
Long-range electrostatic energy as a function of CV_dist(CM-CM)_. Nonbonded interaction energy, computed as electrostatic and Lennard-Jones terms, was evaluated along with the simulation as a function of the distance between the centers of mass of the peptide and H-2D^b^ (CV_dist(CM-CM)_). CV_dist(CM-CM)_ was measured in angstrom and nonbonded energy in kcal/mol. Vertical dotted lines indicate the limits of the regions R_1_, R_2_, and R_3_. Region R_1_ (8 Å < CV_dist(CM − CM)_ ≤ 18.5 Å) with the lowest values for nonbonded energy, which the encounter of the peptide and H-2D^b^ can evolve to form a stable complex. Region R_2_ (18.5 Å < CV_dist(CM − CM)_ ≤ 27.5 Å), in which the distance between the peptide and the receptor is suitable to form a precomplex. Region R_3_ (CV_dist(CM − CM)_ < 27.5 Å), with a site-to-site communication with almost no influence at all.

One more aspect to consider about the interaction between the peptide and the H-2D^b^ receptor is the analysis of the long-range electrostatic energy as a function of the distance between the centers of mass of the peptide and H-2D^b^, i.e., the CV_dist(CM-CM)_. Our analysis suggested a minimum distance between the ligand and receptor in which the long-range electrostatic attraction can promote an association of them. Our analysis detected three regions of interaction for nonbonded energy. The first region (labelled R_1_ in Figure 7), with the lowest nonbonded energy values, corresponds to a distance between the peptide and H-2D^b^ of up to 18.5 Å. In this region, the nonbonded energy can promote the association of the peptide and the receptor. The second region (labelled R_2_ in Figure 7) shows that the interaction intensity was reduced to values higher than the median of nonbonded energy obtained for the first region (-347.72 kcal/mol). In the second region, contacts between the H-2D^b^ protein and the peptide occur to form a precomplex. In this region, molecular diffusion plays a decisive role for binding, driving the complex formation process [45]. Finally, the third region (labelled R_3_ in Figure 7) is where no influence of nonbonded energy was observed, with the intermolecular interaction energy approaching zero (less than 10% of the initial intermolecular interaction energy, i.e., -47.63 kcal/mol). Therefore, beyond the limit of 27.5 Å, no influence of the H-2D^b^ receptor over the peptide TT830-843 was observed.

**Figure 8:**
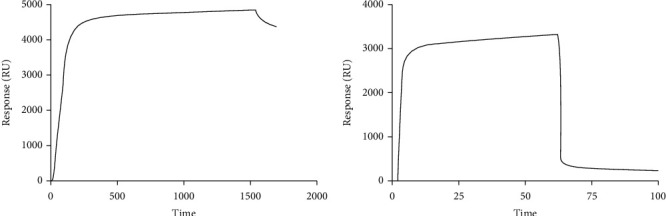
Sensorgrams of the real-time interaction of tetanus toxin peptide and DimerX. DimerX protein immobilization (0.1 *μ*g/*μ*L) on the gold sensor chip COOH5 (a) was performed by EDC/NHS crosslinking of carboxylates to the primary amine procedure onto the sensor chip of the DimerX Fc region. Immobilization step was followed by 240 seconds to achieve the maximum sensor chip cover. Peptide interactions (b) were conducted at 37°C in a final volume of 100 *μ*L of running buffer (PBS, pH 7.4 containing 0.1% DMSO). These data indicate the triplicate variation of the resonance during the kinetics of peptide interaction (31 *μ*M) in running buffer followed by 600 seconds. Data were analyzed by subtracting the reference line using QDAT software. These data represent signal response values in resonance units (RU) versus time in seconds (s) of three independent experiments.

Binding simulation between the TT830-843 epitope with the H-2D^b^ protein assessed by *in silico* assay was confirmed by *in vitro* assay (see Figure 8). Real-time interactions were measured by surface plasmon resonance methodology, showing the parameters for complex formation. Kinetics of the interaction between DimerX and the peptide on the sensor chip after immobilization of the complexes was evaluated by two consecutive applications of the SensiQ Pioneer biosensor, an initial injection of the DimerX protein and a final injection of the peptide. DimerX was bound to the COOH5 chip by the Fc region and exhibited an average binding rate of 4548 RU/s (Figure 8(a)). Therefore, the *α*1 and *α*2 domains of the H-2D^b^ protein were free to interact with the peptide in solution, as previously shown to identify H-2 epitopes and detect specific CD8^+^ T-lymphocytes in the immune response of *Leishmania* (*Leishmania*) *amazonensis* cysteine proteinase B [46, 47].

Interactions between the peptide and H-2D^b^ were performed at pH 7.2 at the temperature of 37°C. They showed 6.7 ± 0.5 × 10^3^ RUmax values in three round assays (Figure 8(b)). Receptor-ligand binding kinetics were also accessed to determine the association (*k*_*a*_ = 9 ± 1 × 10^5^ M^−1^s^−1^) and dissociation (*k*_*d*_ = 0.31 ± 0.02 s^−1^) rate constants, being possible to determine the equilibrium constant as *K*_*D*_ = 344 ± 60 nM. Results of SPR assays showed the reaction between immobilized H-2D^b^ and the epitope follows pseudo-first-order kinetics since the ligand concentration is constant, and the analyte concentration is in excess [48–50].

## 4. Discussion

Peptide TT830-843 is a universal epitope used as a control and adjuvant in acquired immunity studies. This peptide's immunological properties should be a subject of fine molecular interaction studies since it may reveal the nuances of the protective efficiency of the immune response. This work showed the relevant structural and kinetic aspects of this linear epitope necessary to interact with H-2D^b^ proteins.

Exploring the peptide unbinding pathway from the H-2D^b^ receptor and reconstructing the FES assigned to this process, relevant aspects of the mechanisms involved in the peptide exiting were exhibited. Reconstructed FES reveals two distinct energy minima within the bound region, corresponding to different binding modes, labeled as A and B in Figure 4. During the transition from basins A to B, the peptide was exposed to continuous rearrangements seeking favorable interactions. These conformational rearrangements resulted in a protein-ligand complex with a tighter binding conformation. It allowed the complex to accommodate the peptide binding and led the peptide to adopt a low-energy conformation alternative state (~1.15 kcal/mol lower in energy). At this stage, the peptide's central part bulged out until finding a favorable pose. Peptide length can explain this movement. This behavior was already reported for other haplotypes, assigning the peptide length to unpredictably find the exact binding mode for a peptide 14 aa in length [51]. A PDB entry in which the peptide is 11 aa long was used as a scaffold structure to build the complex; therefore, in the first step of WTMetaD, the system sought for a more favorable peptide initial position within the cleft.

In basin B (corresponding to the docked position), H-bond interactions formed between N-terminus peptide residue P1 and residues GLU63, LYS66, TYR159, and TRP167 stabilized this end of the bound peptide. Those H-bond interactions were previously described in the literature [31, 52, 53]. In particular, the influence of residue GLU63 on peptide binding is a key point to understand the effects of inducing cytokines to promote a T-cell response. GLU63 carboxylic moiety and the peptide P1 residue formed a stable H-bond interaction, indicating GLU63 plays an important role in binding affinity. Experimentally, GLU63 was also reported as a relevant residue for peptide binding, inducing the expression of specific cytokines that influence the balance between types of T-cell responses [54]. A second anchor occurred at residue P2, forming H-bond interactions with residues GLU9, GLU63, LYS66, and TYR159. Atoms of the P2 residue formed a persistent H-bond interaction with the polymorphic glutamic acid GLU9 of the beta-sheet floor in the H-2D^b^ receptor. Overall, WTMetaD revealed a persistent H-bond interaction network between peptide residues P1 and P2 and glutamic acid residues in the receptor's groove. It suggests a functional role for glutamic acid residues in the cleft in TT830-843 peptide binding, notably GLU63 and GLU9.

The most persistent H-bond interaction occurred between the C-terminus peptide residue P14 and the *α*2-helix residue LYS146. Undocking pathway was completed only after the disruption of this interaction. Therefore, C-terminus residue P14 was the most important anchor for peptide binding, being a critical component along the undocking pathway.

A transition mechanism for the peptide dissociation was identified. Our analysis of the unbinding trajectory showed that the peptide rested in some stable state. The reconstructed FES consists of five basins, within which there are some transition events connected by transition states. During the transition from basins A to B, the peptide's central part bulged flexibly out of the groove to compensate for length effects. Basin B corresponds to the peptide bounding state. In this state, the peptide stabilized, forming a persistent network of H-bond interactions. Basin C corresponds to an intermediary state visited by the peptide exiting from the cleft. Basin C was visited just after the opening of the *α*-helices, which permitted the dissociation of the peptide's central part but maintained some important H-bond interactions with H-2D^b^. Therefore, the peptide's N- and C-termini anchored the binding to the receptor, with its central part bulged out of the groove. In basin D, only the C-terminus residue P14 anchored the peptide, maintaining H-bond interactions only with the H-2D^b^*α*2-helix. In basin E, the distance between the centers of mass of the peptide and H-2D^b^ indicated that the peptide achieved a fully hydrated state.

WTMetaD simulation provided an approach to reconstruct the underlying FES for the peptide unbinding process, which allowed deriving relevant details about this energetic profile (see Figure 5). In FES, the global energy minimum (basin B) corresponds to the docked state at ~11 Å for CV_dist(CM-CM)_. Transition from basins A to B had to overcome a barrier of ~3 kcal/mol, seeking a tighter peptide binding conformation. After sampling the global minimum, the simulation had to overcome three energy barriers, totalizing ~12 kcal/mol. The simulation overcame these barriers, escaping from basin B. It permitted the opening of the *α*-helices to allow the dissociation of the peptide's central part (transition from basins B to C). After sampling basin C, the simulation reached basin D, the last step of the dissociation process. Finally, the peptide had to pass the last barrier to escape from basin D and fall into the unbound state E. Therefore, the basins were separated by free energy barriers, which were larger than the thermal energy at 310 K (*k*_*B*_*T* = 0.62 kcal/mol). It implies that each barrier crossing would take a time scale inaccessible running a standard MD simulation. The mean of the free energy of dissociation (Δ*G*_diss_ = 8.23 kcal/mol) indicated an external driving force should be applied to dissociate the peptide from the receptor. Therefore, the complex peptide/H-2D^b^ is thermodynamically stable.

In silico mutations were introduced using the psfgen plugin of VMD [34] to evaluate the effects of the substitution of P14 for ALA and GLY residues in WTMetaD simulation. The alanine mutation resulted in a loss of the H-bond association rate with LYS146, but no substantial affinity changes relative to the whole dissociation energy between the peptide and H-2D^b^ were observed (Δ*G*_diss_ = 0.58 kcal/mol). This effect of alanine substitution retains the beta carbon and introduces a small mutant side chain for the P14 residue. This mutation increased the number of H-bond interactions formed between backbone residues of mutant P14 alanine and the cleft of H-2D^b^ (see Table S3 in Supplementary file 1). The glycine mutation removes the beta carbon and can cause flexibility and conformational changes in the peptide. We observed an absence of highly persistent H-bound interactions between the mutant P14 glycine and the H-2D^b^ F pocket, mainly due to the loss of persistence with LYS146 (see Table S4 in Supplementary file 1). It suggests a negative impact on the peptide/H-2D^b^ complex stability and can explain the lower free energy of dissociation estimated for this mutant peptide (Δ*G*_diss_ = 5.16 kcal/mol).

Interaction kinetics are important to analyze (un)binding processes [45], and the half-life factors can help to examine the role of some peptide residues. Kinetic analysis introduces the time component of the observation and reveals other aspects of the interaction between the ligand and its receptor. For instance, in drug discovery research, the kinetic of the drug-receptor binding process can be as important or even more important than affinity in determining drug efficacy, particularly when the pharmacological duration effect is a significant component of *in vivo* efficacy [45, 55–57]. A kinetic analysis was retrieved from the values of half-life factors (described in Figure 6) and the persistence of H-bond interactions in Table 1. Such analysis examined the role of some residues to maintain the peptide binding. It is related to the time during which the peptide remains bound. In Figure 6, the half-life factors are the highest for N- and C-termini, indicating that these regions play an important role in the unbinding trajectory. Specifically, the C-terminus residue P14 has the highest half-life factor and has the longest persistence of H-bond interactions (Table 1). Therefore, it suggests an important role of this residue to drive peptide binding, mainly when interacting with residue LYS146 in the cleft. Standard *in vitro* or *in vivo* assays cannot identify the peptide residues' atomic rearrangements within the cleft and their interaction kinetics. The approach applied in this work allowed to simulate the peptide dissociation on computationally tractable time scales. Therefore, it can be a useful method to observe the interaction kinetics and, consequently, shed light on the molecular mechanism of the peptide exiting. We hypothesize that residues with longer residence time on the receptor are kinetically decisive for peptide binding.

Protein-protein binding sites can reveal an amino acid residue network that communicates structurally and energetically with one another. This site-to-site communication contributes to protein binding, and interactions over long distances provide long-range communication between such binding sites [58]. For binding to occur, the approximation of the two biomolecules should happen at a suitable distance and orientation, for which the molecular diffusion acts on both the ligand and the receptor to form a precomplex. A precomplex is defined as a transitional state necessary for the binding of two molecules. Long-range electrostatic energy can promote this encounter, and it can evolve to a stable state to form a complex [59, 60].

In previous work, our results suggested a behavior pattern for peptides that bind H-2 receptors [31]. Those results were obtained assuming a preset condition for the parameters used to perform enhanced sampling simulations. Computing the nonbonded energy between the peptide and H-2D^b^, three interaction regions were detected. Our results indicated that an encounter between the peptide and the H-2D^b^ receptor can happen at a distance ~27.5 Å to form a precomplex. Within this distance, molecular diffusion can favor the peptide binding, and the process can evolve to form a stable complex. We suggest that this feature can be part of the peptide loading mechanism that occurs in endosomes for cross-presentation. In this case, peptide loading is considered a diffusional step with an entropic barrier due to the penalty associated with peptide rotational and translational entropy loss. Within a certain distance, the peptide can overcome entropic barriers and form a diffusional encounter complex that evolves to bind the receptor.

In SPR assays, although the kinetic findings are in accordance with predicting good protein-protein binding affinity [61], further comments are needed. SPR assay conditions do not reflect the cellular physiochemical conditions in which these interactions occur. However, the measured *k*_*a*_ value indicates that the peptide can form a complex with the H-2D^b^ protein in antigen-presenting cells during the immune response. This fast and spontaneous binding can be one reason for preserving this fragment from complete enzymatic hydrolysis by the proteasome system. Low *k*_*d*_ values found indicate that the peptide maintains a more stable complex with the H-2D^b^ protein to persist long enough to interact with T-lymphocytes.

Additionally, as the recombinant H-2D^b^ protein is supplemented with recombinant beta2-microglobulin (*β*2M), SPR data can be related to the supramolecular complex stability for T-lymphocyte recognition by this epitope [62]. An important experimental consideration for these assays was the Gibbs free energy of the formed complex: ΔG = −8.3 kcal/mol. ΔG negative values indicated spontaneous binding for supramolecular complex formation under pH 7.2 and 37°C. Therefore, the peptide/H-2D^b^ complex presented enough free energy to interact with a TCR. Estimated energy suggests the complex should be stable on the antigen-presenting cell surface, whose pH is close to neutral, as described for epitopes from the C-terminus extension of cysteine proteinase B from *L.* (*L.*) *amazonensis* [46].

## 5. Conclusion

Using an *in silico* method, some key residues for peptide binding to H-2D^b^ receptors were identified. Glutamic acids in the cleft, notably GLU9 and GLU63, play an essential role in peptide binding. It also pointed out that the C-terminus residue P14 is the most important anchor for the peptide interaction with the H-2D^b^ receptor.

During the simulation, the exiting from the cleft is characterized by a stepwise mechanism between progressively detached states until the full dissociation of the peptide. An important functional feature for peptide detachment was observed: the opening of *α*-helices to permit the peptide's central part dissociates from the cleft. Additionally, some relevant interactions that occurred during the dissociation process were described: in particular, the H-bond interactions occurred between the receptor and C- and N-terminus peptide residues. These unbinding pathway functional features were detected running a relatively short simulation. This *in silico* assay was crucial to describe important functional features, which has been impossible to investigate using experimental techniques. Therefore, we assumed WTMetaD as a valuable method to confirm interactions among multiple residues of a protein complex. This method can be used to investigate binding contacts that have a functional implication.

The approach applied in this work helped to understand the peptide dissociation mechanistic basis and indicate whether a complex is a thermodynamically stable system. To date, we do not know any experimental results of calculating the free energy of dissociation for complexes formed between MHC class I receptors and 14 residue-long peptides. Therefore, although previous work has used this approach to calculate and compare the free energy of dissociation for 8-10 amino acid peptides in complexes with MHC class I [31], further studies should be conducted to evaluate the effect of longer peptides in determining the free energy of dissociation in complexes with 14 residue-long peptides.

FES projection onto the CV_dist(CM-CM)_ revealed the energetic profile of the peptide exiting, providing an understanding of how the unbinding process overcame the surface's energy barriers.

We suggested a minimal distance between the peptide and the H-2D^b^ receptor to favor the peptide binding. We can understand this feature as a diffusional step that can be part of the peptide loading mechanism, in which the peptide must overcome an entropic barrier to load into the receptor. Future work can also consider this feature to figure out the peptide presentation to the immune system and how they compete to bind to MHC class I molecules.

*In silico* assays were confirmed by additional surface biosensing experiments that use the DimerX (H-2D^b^ haplotype). Data gathered in these assays reinforce that the peptide can form a stable complex with H-2D^b^. This complex exhibits enough free energy to interact with a TCR on the antigen-presenting cell surface. Therefore, the DimerX used in SPR assays in combination with WTMetaD simulation indicates a promising approach to study real-time interactions between ligands and receptors. In our specific case, the combination of *in silico* and *in vitro* assays provided significant evidence supporting the formation of a stable complex between the peptide TT830-843 and H-2D^b^ receptor.

## Figures and Tables

**Table 1:** Selected H-bond interaction persistence.

Peptide residue^∗^	H-2D^b^ residue	% ^∗∗^
GLN P1	GLU63	12.09
GLN P1	LYS66	2.69
GLN P1	TYR159	4.78
GLN P1	TRP167	7.29
TYR P2	GLU9	8.18
TYR P2	GLU63	5.38
TYR P2	LYS66	4.06
TYR P2	TYR159	8.14
LYS P4	GLU163	3.24
ASN P6	HSE155	2.58
GLU P14	SER77	2.23
GLU P14	ASN80	2.51
GLU P14	LYS146	15.81
GLU P14	SER150	2.64

^∗^Peptide residues are named as P1 (N-terminus) and P14 (C-terminus). ^∗∗^Considering only persistence corresponding to the percentage of the total number of H-bond interactions greater than 2%. This column provides information for 81.62% of the H-bond interactions that occurred during WTMetaD simulation. Completed table containing 100% of the H-bond interactions is available in Table S1 of Supplementary file 1.

## Data Availability

All data generated or analyzed during this study are included in this article (and its supplementary information file).

## Supplementary Materials

Supplementary Materials Supplementary Figures S1, S2, and S3; Supplementary Tables S1, S2, S3, and S4. Supplementary file 2: side view of the peptide exiting trajectory from the H-2D^b^ protein's cleft during WTMetaD simulation. Supplementary file 3: top view of the peptide exiting trajectory from the H-2D^b^ protein's cleft during WTMetaD simulation. Supplementary file 4: a gif animated image showing the projection of the FES plot of the configurations sampled during WTMetaD.
