# Supplementary material for: Combining Well-Tempered Metadynamics Simulation and SPR Assays to Characterize the Binding Mechanism of the Universal T-Lymphocyte Tetanus Toxin Epitope TT830-843
Source: Biomed Res Int. 2021 Jul 4;2021:5568980. doi: 10.1155/2021/5568980 (PMC8275407; doi:10.1155/2021/5568980)
Supplement: Supplementary Materials — Supplementary Figures S1, S2, and S3; Supplementary Tables S1, S2, S3, and S4. Supplementary file 2: side view of the peptide exiting trajectory from the H-2Db protein's cleft during WTMetaD simulation. Supplementary file 3: top view of the peptide exiting trajectory from the H-2Db protein's cleft during WTMetaD simulation. Supplementary file 4: a gif animated image showing the projection of the FES plot of the configurations sampled during WTMetaD. [file 5568980.f1.pdf]

BioMed Research International

Title

Combining well-tempered metadynamics simulation and SPR assays to characterize the binding mechanism of the universal T-lymphocyte tetanus toxin epitope TT830-843

Artur A. M. L. Brandt[1,6], Rodrigo N. Rodrigues-da-Silva[2], Josué C. Lima-Junior[3], Carlos R. Alves[4,*], Franklin de Souza-Silva[5,7]

[1]Faculdade de Educação Tecnológica do Estado do Rio de Janeiro, Rua Clarimundo de Melo, 847, CEP 21311-281, Rio de Janeiro, RJ, Brasil.

[2]Fundação Oswaldo Cruz, Instituto de Tecnologia em Imunobiológicos, Laboratório de Tecnologia Diagnóstica, Avenida Brasil, 4365, CEP 21040-900, Rio de Janeiro, RJ, Brasil.

[3]Fundação Oswaldo Cruz, Instituto Oswaldo Cruz, Laboratório de Imunoparasitologia, Avenida Brasil, 4365, CEP 21040-900, Rio de Janeiro, RJ, Brasil.

[4]Fundação Oswaldo Cruz, Instituto Oswaldo Cruz, Laboratório de Biologia Molecular e Doenças Endêmicas, Avenida Brasil, 4365, CEP 21040-900, Rio de Janeiro, RJ, Brasil.

[5]Centro de Desenvolvimento Tecnológico em Saúde, Fundação Oswaldo Cruz, Rio de Janeiro, Brasil.

[6]Univeritas-Rio, Rua Marques de Abrantes, 55, CEP 2230-060, Rio de Janeiro, RJ, Brasil.

[7]Universidade Iguaçu, Faculdade de Ciências Biológicas e da Saúde, Rio de Janeiro, RJ, Brasil

*Corresponding author E-mail adresses: artur.brandt@faeterj-rio.edu.br (A.A.M.L. Brandt), rodrigonunes.ioc@gmail.com (R.N.Rodrigues-da-Silva), josue@ioc.fiocruz.br (J.C. Lima-Junior), calves@ioc.fiocruz.br (C.R.Alves), franklin.frankss@gmail.com (F.Souza-Silva)

Supplementary information

File name: Supplementary _file_1.pdf.

File format: pdf.

Title of data: Supplementary figures and tables.

Description of data: Supplementary figures S1, S2, S3, and S4; Supplementary tables S1, S2, S3, and S4.

File name: Supplementary_file_2.mov.

File format: mov.

Title of data: Peptide exiting from H-2D^b (side view).

Description of data: Side view of the peptide exiting trajectory from the H-2D^b protein's cleft during WTMetaD simulation.

Link to the file: https://drive.google.com/file/d/1j7c--X3t8f0zQjL0DrP3AKYLclyJv0Ei/view?usp=sharing

File name: Supplementary_file_3.mov.

File format: mov.

Title of data: Peptide exiting from H-2D^b (top view).

Description of data: Top view of the peptide exiting trajectory from the H-2D^b protein's cleft during WTMetaD simulation.

Link to the file: https://drive.google.com/file/d/1-jYqKvJAHyNssplAD9re-ojw-rYSuHB8/view?usp=sharing

File name: Supplementary_file_4.gif.

File format: gif.

Title of data: Animated image of the configurations sampled during WTMetaD.

Description of data: A gif animated image showing the projection onto the FES plot of the configurations sampled during WTMetaD

Link to the file: https://drive.google.com/file/d/1acf2iB5wNbqDM5RPYZyv0K8sEannRlSs/view?usp=sharing

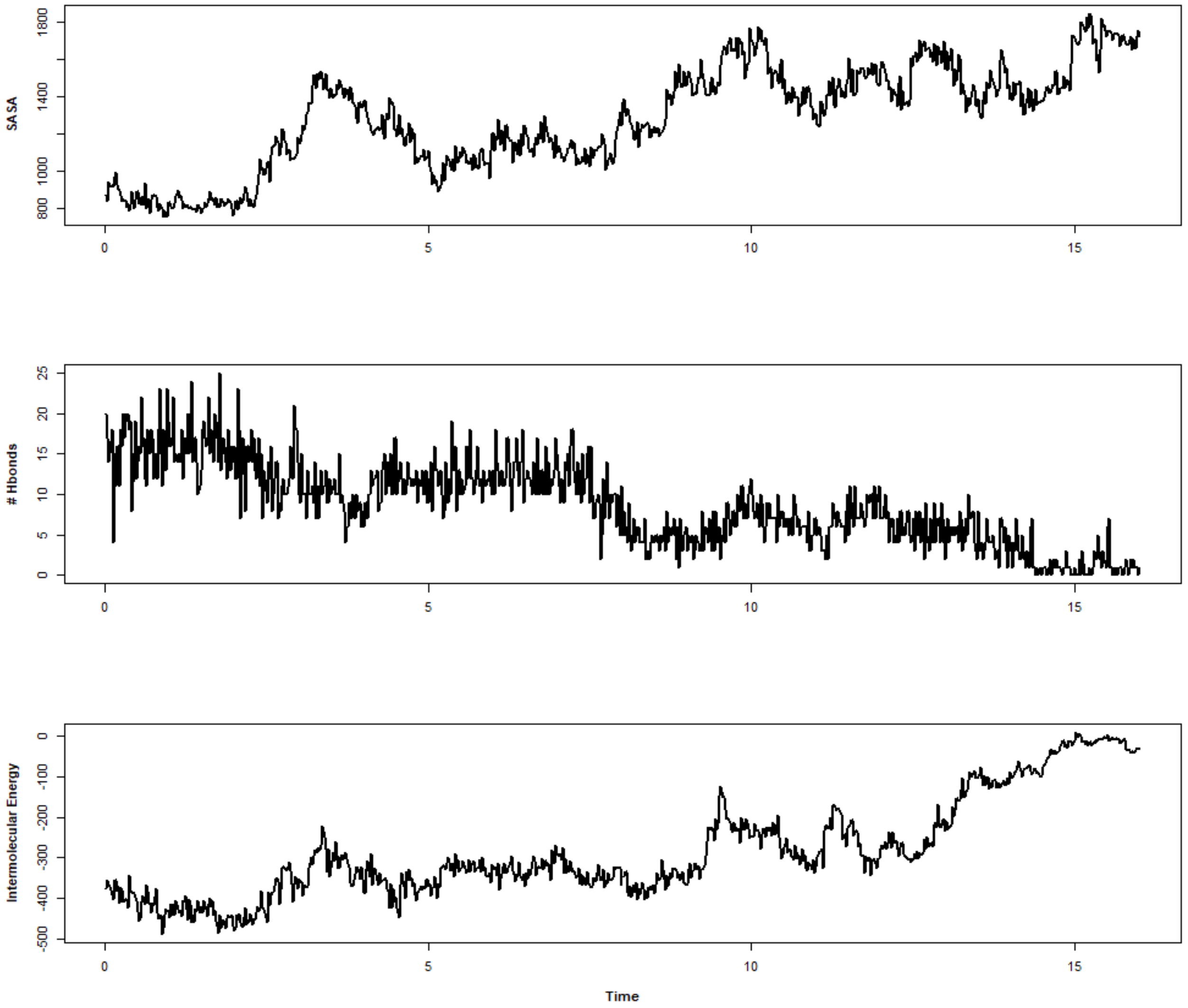

Fig. S1 Time evolution for surface accessible solvent area (SASA), number of hydrogen bonds (#Hbonds), and intermolecular interaction energy (Intermolecular Energy). “SASA” is measured in Å^2. “#Hbonds” corresponds to the sum of all hydrogen bonds between the peptide and H-2D^b receptor in each simulation frame. “Intermolecular Energy” is measured in kcal/mol as the sum of electrostatic and Lennard-Jones terms. In all panels, structure properties were plotted as function of simulation time in nanosecond (ns).

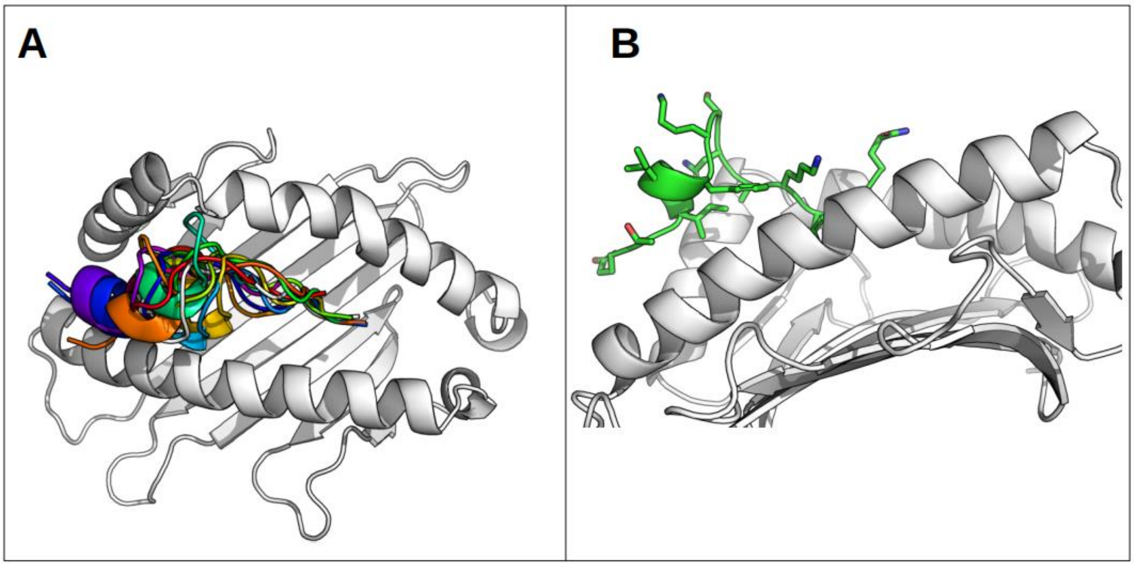

Fig. S2 Results for job submitted to FlexPepDock webserver. (A) Resulting models obtained from refinement of coarse starting model. Each model is represented in different colors. (B) Best-ranked pose, the structure with the lowest energy used as the initial structure for WTMetaD simulation.

The stability of the peptide initial molecular structure

A minimization-equilibration cycle was performed to prevent the introduction of artifacts from an unstable starting structure obtained from Rosetta FlexPepDock and to ensure that the positions of peptide initial molecular configuration are reasonable. This step was used to validate whether the peptide structure is conformationally stable. Fig. S3 plots the calculated peptide backbone RMSD (root mean square deviation) to assess its stability within the H-2 D^b receptor. Calculated RMSD converges to an average value of 1.85 Å, meaning the system was equilibrated before the production simulation.

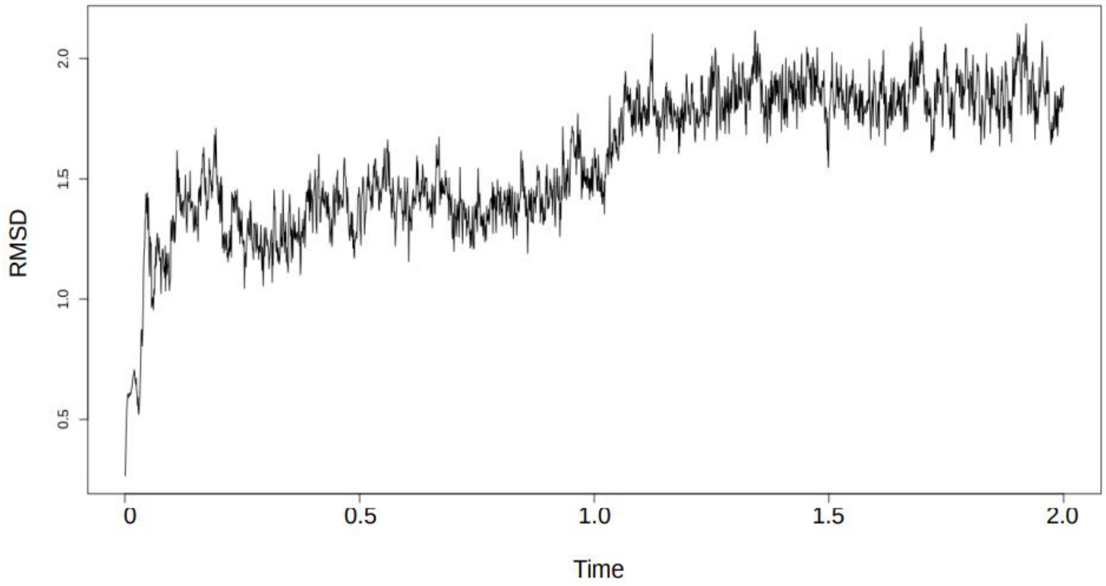

Fig. S3 The stability of the peptide initial molecular structure. Peptide backbone RMSD versus simulation time during minimization-equilibration cycle. RMSD is measured in Å and simulation time in nanosecond (ns).

Table S1 Persistence of H-bond interactions during WTMetaD simulation.

Peptide residue	H-2D^{b} residue	%
GLN P1	TYR 7	0.01
GLN P1	GLU 58	0.04
GLN P1	TYR 59	1.51
GLN P1	ARG 62	1.54
GLN P1	GLU 63	12.09
GLN P1	LYZ 66	2.69
GLN P1	TYR 159	4.78
GLN P1	GLU 163	0.04
GLN P1	TRP 167	7.29
GLN P1	TYR 171	0.13
TYR P2	TYR 7	0.52
TYR P2	GLU 9	8.18
TYR P2	SER 24	0.01
TYR P2	GLU 63	5.38
TYR P2	LYZ 66	4.06
TYR P2	GLN 70	1.38
TYR P2	TRP 73	0.02
TYR P2	GLN 97	1.43
TYR P2	SER 99	0.69
TYR P2	HSE 155	0.15
TYR P2	TYR 156	0.33
TYR P2	TYR 159	8.14
TYR P2	GLU 163	0.03
TYR P2	TRP 167	0.01
ILE P3	LYS 66	1.29
ILE P3	GLN 70	0.05
ILE P3	TRP 73	0.01
ILE P3	GLU 154	0.01
ILE P3	HSE 155	1.21
ILE P3	TYR 156	0.53
ILE P3	ALA 158	0.28
ILE P3	TYR 159	1.17
ILE P3	GLU 163	0.17
LYS P4	GLN 65	0.01
LYS P4	LYZ 66	0.59
LYS P4	HSE 155	0.02
LYS P4	GLU 163	3.24
LYS P4	GLU 166	0.79
ALA P5	ARG 62	0.04
ALA P5	LYZ 66	0.01
ALA P5	GLN 70	0.14
ALA P5	TRP 73	0.01
ALA P5	HSE 155	0.31
ASN P6	ARG 62	0.26
ASN P6	LYZ 66	0.25
ASN P6	GLU 154	0.04
ASN P6	HSE 155	2.58
ASN P6	ALA 158	0.04
SER P7	GLU 58	0.03
SER P7	ARG 62	0.05
SER P7	GLN 72	0.03
SER P7	TRP 73	0.05
SER P7	SER 150	0.02
SER P7	GLU 154	0.02
SER P7	HSE 155	0.71
LYS P8	TRP 73	0.13
LYS P8	SER 150	0.19
LYS P8	GLY 151	0.21
LYS P8	HSE 155	0.08
PHE P9	LYZ 66	0.01
PHE P9	GLN 72	0.54
PHE P9	TRP 73	0.41
PHE P9	VAL 76	0.21
PHE P9	HSE 155	0.01
PHE P9	ALA 158	0.02
PHE P9	GLU 163	0.02
ILE P10	GLN 72	0.06
ILE P10	VAL 76	0.03
ILE P10	ARG 79	0.01
ILE P10	LYS 146	0.05
ILE P10	HSE 155	0.01
GLY P11	LYS 146	0.04
GLY P11	SER 150	0.02
ILE P12	ARG 62	0.03
ILE P12	GLY 69	0.05
ILE P12	GLN 72	0.19
ILE P12	TRP 73	1.17
ILE P12	VAL 76	0.02
ILE P12	ALA 152	0.08
ILE P12	HSE 155	0.04
ILE P12	TYR 156	0.07
THR P13	TRP 73	1.31
THR P13	VAL 76	0.39
THR P13	SER 77	0.06
THR P13	ASN 80	0.09
THR P13	LYS 146	0.03
THR P13	TRP 147	1.22
GLU P14	TRP 73	0.03
GLU P14	SER 77	2.23
GLU P14	ASN 80	2.51
GLU P14	TYR 84	0.29
GLU P14	TYR 123	1.07
GLU P14	THR 143	0.32
GLU P14	LYS 146	7.81
GLU P14	TRP 147	1.89
GLU P14	SER 150	2.64
Total %		100

Table S2 FlexPepDock top 10 models

Description	Total score (*)	RmsBB (Å)
Top 1	-108.304	6.932
Top 2	-107.106	2.151
Top 3	-106.810	4.392
Top 4	-104.485	3.509
Top 5	-104.362	2.407
Top 6	-103.209	2.832
Top 7	-100.851	5.914
Top 8	-100.680	3.442
Top 9	-100.406	3.908
Top 10	-100.189	5.865
ALA P14	TYR 84	7.11
ALA P14	THR 143	9.19
ALA P14	LYS 146	6.98

(*) The models obtained from FlexPepDock were ranked according to Rosetta energy score.

Table S3 Selected H-bond interaction persistence during WTMetaD for ALA mutation in the peptide's position P14.

Peptide residue	H-2D^b residue	% (*)
GLN P1	GLU 163	3.59
GLN P1	TRP 167	4.15
TYR P2	GLU 9	3.30
TYR P2	GLU 63	3.73
ILE P3	HSE 155	2.14
ILE P10	SER 150	2.07
ILE P12	TRP 73	3.17
THR P13	TRP 147	6.42
ALA P14	SER 77	6.60
ALA P14	ASN 80	5.16
ALA P14	TYR 84	7.11
ALA P14	THR 143	9.19
ALA P14	LYS 146	6.98

(*) Considering only persistence corresponding to the percentage of the total number of H-bond interactions greater than 2 %.

Table S4 Selected H-bond interaction persistence during WTMetaD for GLY mutation in the peptide's position P14.

Peptide residue	H-2D^b residue	% (*)
GLN P1	TYR 59	2.95
GLN P1	GLU 63	11.57
GLN P1	TYR 159	2.85
GLN P1	TRP 167	5.41
TYR P2	GLU 9	3.26
TYR P2	LYS 66	2.37
TYR P2	GLN 70	2.22
TYR P2	HSE 155	2.47
TYR P2	TYR 159	5.40
TYR P2	GLU 163	2.21
LYS P4	GLU 163	10.19
LYS P4	GLU 166	3.90
LYS P8	HSE 155	2.01
THR 13	TRP 147	2.09
GLY P14	ASN 80	2.30
GLY P14	THR 143	2.73
GLY P14	LYS 146	2.23

(*) Considering only persistence corresponding to the percentage of the total number of H-bond interactions greater than 2 %.
